# Exploratory Study of the Frequency of Detection and Tissue Distribution of *Porcine Circovirus 3* (PCV-3) in Pig Fetuses at Different Gestational Ages

**DOI:** 10.3390/pathogens11020118

**Published:** 2022-01-20

**Authors:** Albert Ruiz, Viviane Saporiti, Eva Huerta, Mònica Balasch, Joaquim Segalés, Marina Sibila

**Affiliations:** 1 Zoetis Manufacturing & Research Spain S.L., Ctra. Camprodon s/n, La Riba, 17813 Girona, Spain; albert.ruiz.llombart@outlook.com (A.R.); monica.balasch@zoetis.com (M.B.); 2 IRTA, Centre de Recerca en Sanitat Animal (CReSA, IRTA-UAB), Campus de la Universitat Autònoma de Barcelona, 08193 Barcelona, Spain; viviane.saporiti@irta.cat (V.S.); eva.huerta@irta.cat (E.H.); 3 OIE Collaborating Centre for the Research and Control of Emerging and Re-Emerging Swine Diseases in Europe (IRTA-CReSA), 08193 Barcelona, Spain; joaquim.segales@irta.cat; 4 Departament de Sanitat i Anatomia Animals, Universitat Autònoma de Barcelona (UAB), 08193 Barcelona, Spain; 5 Centre de Recerca en Sanitat Animal (CReSA, IRTA-UAB), Campus de la Universitat Autònoma de Barcelona, UAB, 08193 Barcelona, Spain

**Keywords:** *porcine circovirus 3* (PCV-3), reproductive failure, aborted fetuses, stillborn, quantitative PCR, subclinical infection

## Abstract

*Porcine circovirus 3* (PCV-3) has been associated with several pig diseases. Despite the pathogenicity of this virus has not been completely clarified, reproductive disorders are consistently associated with its infection. The aim of the present work was to analyze the presence of PCV-3 DNA in tissues from pig fetuses from different gestational timepoints. The fetuses were obtained either from farms with no reproductive problems (NRP, *n* = 249; all of them from the last third of gestation) or from a slaughterhouse (S, *n* = 51; 49 of the second-third of gestation and 2 from the third one). Tissues collected included brain, heart, lung, kidney, and/or spleen. Overall, the frequency of detection of PCV-3 was significantly higher in fetuses from the last third of the gestation (69/251, 27.5%) when compared to those from the second-third (5/49, 10.2%), although the viral loads were not significantly different. Moreover, the frequency of detection in NRP fetuses (69/249, 27.7%) was significantly higher than in S ones (5/51, 9.8%). Furthermore, PCV-3 DNA was detected in all tissue types analyzed. In conclusion, the present study demonstrates a higher frequency of PCV-3 DNA detection in fetuses from late periods of the gestation and highlights wide organ distributions of the virus in pig fetuses.

## 1. Introduction

*Porcine circoviruses* (PCVs) are icosahedral, non-enveloped viruses characterized by a circular single-stranded DNA belonging to the *Circoviridae* family [1]. To date, four different species of PCVs have been described and named numerically after their chronological discovery, PCV-1, PCV-2, PCV-3, and PCV-4. PCV-1 was described in 1974 and is regarded as a non-pathogenic virus for swine [2,3]. PCV-2 is one of the most economically important viruses affecting the swine industry, basically due to the mortality rate associated with it and the growth retardation caused in postweaning piglets [4]. PCV-2 associated diseases (PCVD) comprise a range of different conditions, including PCV-2-systemic disease, PCV-2-reproductive disease, porcine dermatitis, and nephropathy syndrome (PDNS) [4]. PCV-3 is currently being investigated as a potential pathogen in different clinical conditions, mainly in relation to cases of reproductive problems and multisystemic lymphoplasmacytic inflammation in pre- and post-weaning pigs [5,6]. Indeed, it has been recently proposed that the so-called PCV-3 associated diseases (PCV-3-AD) may include two main disorders: PCV-3 reproductive disease (PCV-3-RD) and PCV-3 systemic disease (PCV-3-SD) [7]. Finally, PCV-4 was identified in 2019 in pigs with clinical conditions, such as PDNS, digestive signs [8], respiratory disorders [9], and neurological problems [10], but little information is available regarding its pathogenicity; its importance in the porcine industry is unknown. Moreover, the virus has thus far not been detected in other parts of the world, but Asia [11].

PCV-3 was firstly identified in tissue samples from animals with PDNS-like signs, increased mortality, and reproductive failure cases characterized by an increase in mummified and stillborn fetuses. In addition, the viral genome was found in piglets with myocarditis and systemic vascular inflammation [12,13]. PCV-3 DNA has also been detected in animals displaying different clinical signs, such as respiratory, gastrointestinal, and neurological disorders [13,14]. However, the simple presence of genomic DNA does not imply viral replication and is not enough to establish causality of a disease [7,15]. Indeed, the most convincing proof of PCV-3 pathogenicity to date [6,16] would be the detection of PCV-3 DNA by in situ hybridization (ISH) within lesions of fetuses from reproductive cases (mummified fetuses, late abortions, stillborn, and weak-born piglets). These latter cases would be diagnosed under the scope of the recently proposed PCV-3-RD [7,15]. Moreover, this virus has also been detected in high prevalence in wild boars [17,18,19] and, sporadically, in other wildlife species [20], highlighting the importance of wildlife surveillance to look for potential reservoirs of infectious pathogens or spillover events [21].

Thus far, the solidest evidence of causation is found in relation to PCV-3 causing reproductive problems [6,12,13,16]. In 2018, a Brazilian group reported the detection of PCV-3 in serum samples from sows with variable numbers of stillborn fetuses, whereas sera from sows without stillbirths were PCV-3 negative [22]. In addition, the most recent case of PCV-3 vertical transmission comes from a pig farm in Colombia, where a PCV-3 PCR positive gilt with reproductive problems was followed-up during pre-farrowing, farrowing, and weaning periods; PCV-3 viral loads were high in the placenta and a mummified fetus, and the presence of PCV-3 was detected in pre- and post-colostrum sera of the piglets and in the colostrum itself, demonstrating PCV-3 vertical transmission [23]. Another study indicated that PCV-3 presence in stillborn and mummified fetuses can happen even in farms with normal reproductive parameters [24]. Moreover, a longitudinal study, where the PCV-3 infection dynamics in conventional Spanish farms were analyzed, showed a relatively high number of PCV-3 positive piglets at early ages [25], suggesting that intrauterine infections might play a role in early PCV-3 infection.

Thus far, it seems that PCV-3 intrauterine infections are frequent, even in association with reproductive failure cases [6,16]. However, the exact moment in which fetal susceptibility to PCV-3 infection is higher, as well as the PCV-3 replication sites, remain unclarified. Based on exposed evidence, the aim of the current study was to analyze the presence of PCV-3 DNA in pig fetuses from different gestational timepoints, and to determine which fetal tissues harbor viral genomes.

## 2. Results

### 2.1. Proportion of PCV-3 qPCR Positive Fetuses from Different Gestational Ages

The estimated fetal age average of the fetuses from the second (*n* = 49) and last (*n* = 251) thirds of gestation were 48 ± 11 dg and 108 ± 8 dg, respectively.

Overall, PCV-3 was detected in 74 out of 300 (24.6% CI (19.9–29.9%)) fetuses tested, being quantifiable in 64 of them (Table 1). The proportion of PCV-3 positive samples among fetuses from the second-third of the gestation (5/49, 10.2% CI (3.4–22.2%)) was significantly lower than in the fetuses from the last third of the gestation (69/251, 27.5% CI (22.1–33.5%)) (*p* = 0.010). Differences in viral loads between different gestational ages could not be calculated since only one out of the four fetuses from the second gestational third positive by qPCR had a quantifiable PCV-3 load.

### 2.2. Proportion of PCV-3 Positive Fetuses from Different Origins

The proportion of PCV-3 positive samples of fetuses from group NRP (no reproductive problems) (69/249, 27.7% CI (22.3–33.7%)) was significantly higher (*p* = 0.0069) than the fetuses from the slaughterhouse (group S) (5/51, 9.8% CI (3.3–21.4%)). Differences in viral loads between different groups could not be assessed, due to the small sample sizes of quantifiable PCV-3 positive samples (two samples in S fetuses).

### 2.3. PCV-3 Detection in Fetal Tissues

Individual tissue samples were available from 64 of the PCV-3 qPCR positive fetuses with quantifiable viral loads; two S fetuses and 62 NRP fetuses. All but one of the 64 fetuses corresponded to the last third of gestation. Individual results from tissues are included in Table 2. PCV-3 was not detected in the tissue samples from the only fetus obtained from the second-third of gestation. On the contrary, PCV-3 was detected in all tissue types examined in fetuses from the last third of gestation. In 42 fetuses (65%), all tissues assessed were qPCR positive. No significant differences (*p* = 0.11) regarding the proportion of PCV-3 positive samples or the quantity (*p* = 0.38) of PCV-3 DNA were found between tissue types.

## 3. Discussion

The present study assessed the frequency of PCV-3 detection in fetuses according to their gestational timing (second- versus last third of gestation), as well as its tissue distribution. Despite the role of intrauterine PCV-3 infection has being partially described [16,23,24], the prevalence of PCV-3 throughout fetal age has not been assessed. Here, we report a significantly higher proportion of PCV-3 qPCR positive fetuses within the last third of the gestation (immunocompetent fetuses) when compared to fetuses from the second-third of gestation (pre-immunocompetent fetuses).

The higher prevalence of PCV-3 in fetuses from the last gestational third could be responsible for the potential early and/or long-lasting infections reported in piglets from 2 to 22 weeks of age [25]. On the other hand, PCV-3 infection in the second-third of gestation, due to the lack of fetal immunocompetence, may lead to the birth of immunotolerant pigs. This latter scenario would explain potential persistent PCV-3 infections postnatally, as it has also been sporadically observed in the abovementioned work, in which few animals were PCR positive at all sampling points during their lifespan [19]. However, the current available laboratory techniques do not allow the full assessment of such condition. Similarly, long-lasting infections have been reported in other ssDNA swine viruses, such as PCV-2 [26,27] and Torque teno sus viruses [28,29]. In addition, it is also known that fetal infection can occur at different gestational timepoints with these viruses, with an outcome that depends on the moment when the sow is exposed to the pathogen [4]. Altogether, it is still early to speculate on the persistency of PCV-3 infection in fetuses and the relevance of the fetal immune competency in susceptibility to infection.

In the present work, the number of samples coming from farms without overt reproductive problems was higher than the ones coming from the slaughterhouse. Although this situation is not ideal and may represent a bias in the proportion of positive samples in each group, the results obtained are relevant as an exploratory study since they indicate that PCV-3 intrauterine infections are rather frequent and not necessarily associated to productivity disorders of the sow, expanding the already existing information [24]. However, the obtained results would agree with a recently published study analyzing the frequency of PCV-3 detection in fetuses from reproductive cases [16]. This study described 30.2% of PCV-3 PCR positive cases among pooled fetal tissue samples, being, in all cases, fetuses from the last third of the gestation. That report fits well with the overall assumption that reproductive diseases in sows affecting late gestational periods may result in abortions, stillborn piglets, and/or premature farrowing [6,23].

The results obtained in this study point towards a wide PCV-3 fetal tissue distribution. This is not rare since PCV-3 has been already found in a wide array of fetal organs and throughout the duration of the pregnancy, mainly in highly vascularized tissues, such as the brain [6], lung [6,30], kidney [6], spleen [31], heart [16], and tracheobronchial and mesenteric lymph nodes [23]. In the present study, the highest PCV-3 DNA loads were found in the lung and brain (reaching up to 10^9^ PCV-3 DNA copies/mL) but were not significantly different from those found in the heart, kidney, or spleen tissues. These viral loads would be in line with the existing literature, which shows considerable variability, even in fetuses from the same litter [6,23], and ranged approximately from 10^4^ to 10^11^ PCV-3 DNA copies/mL, depending on the tissue examined. Nevertheless, one should bear in mind that the mere detection of DNA in a sample does not imply replication and, therefore, further studies to assess in which tissues the virus is actively replicating would be needed [15].

## 4. Materials and Methods

A total of 300 fetuses at different stages of the gestation were included in this study. Fetuses were classified in two gestational ages using the crown-to-rump length (CRL) [32]: fetuses from 30 to 69 days of gestation (dg, second-third of the gestation, *n* = 49, 16.3%) and fetuses from 70 to 115 dg (last third of the, *n* = 251, 83.6%). These fetuses came from two different origins. Group NRP included stillborn fetuses from farms without overt reproductive problems (*n* = 249, 83%); this group comprised fetuses from a previously published study [24], with both brain and lung tissues available (*n* = 206) and fetuses collected from different Spanish farms in 2020 and 2021 (*n* = 43). Finally, Group S comprised fetuses collected from sacrificed pregnant sows (from different farms) in a commercial slaughterhouse located in the northeastern part of Spain (*n* = 51, 17%).

From each fetus, a pool with the tissues collected during the necropsy (brain, heart, lung, kidney, and/or spleen) was generated. The number of tissues tested per fetus ranged from 2 to 5. Briefly, approximately 0.5–1 cm^3^ of each individual tissue was cut, manually minced, and mixed with 600 µL of PBS (Lonza, Basel, Switzerland) containing six glass spheres. Afterwards, tissues were homogenized with a TissueLyser II machine (Qiagen, Venlo, Netherlands), programmed at 30 Hz for 10 min. The supernatant from individual tissue samples coming from the same fetus were then pooled together in equal volumes, up to a volume of 200 µL of pooled supernatants.

DNA was extracted from 200 µL of the pooled supernatant using MagMAX^TM^ Pathogen RNA/DNA Kit (Applied Biosystems^®^, Waltham, MA, USA), according to manufacturer’s instructions. A mixture of 180 µL of tissue supernatant and 20 µL of full-length PCV-3 genome plasmid and 200 µL of doubled-distilled water served as positive and negative extraction controls, respectively. Extracted DNA was processed by quantitative real-time PCR (qPCR) to detect PCV-3 genome, as described previously [24,33]. A standard curve containing serial 1:10 dilutions of the full-length PCV-3 genome plasmid was included in the assay to quantify PCV-3 viral loads. The qPCR results were expressed in PCV-3 DNA copies/mL of supernatant of tissue pool homogenate. The quantification limit of the assay was set between 10^2^ and 10^3^ PCV-3 DNA copies/mL of tissue homogenate; positive samples below the limit of quantification (BQL) of the assay were considered non-quantifiable. In case the pool of tissue from one fetus was positive, the supernatant of the different tissue homogenates was, when possible, individually processed following the same procedure, and results were expressed as PCV-3 DNA copies/mL of the supernatant of the individual tissue homogenate.

Frequencies of PCV-3 DNA detection were compared among fetuses from different groups and with different gestational ages using Chi-square or Fisher’s exact test. PCV-3 load between categories, age groups, and tissues were compared using the Kruskal–Wallis test. Statistical analyses were performed with GraphPad (GraphPad software Inc, San Diego, CA, USA). Statistical significance level was set at *p* < 0.05.

## 5. Conclusions

In conclusion, the present work demonstrated a significantly higher frequency of PCV-3 DNA detection in fetuses from the last third of gestation compared to those of the second one and with a wide tissue distribution. Further research is needed to clarify the pathogenesis of fetal infection and replication sites of PCV-3 in the fetuses.

## Figures and Tables

**Table 1:** Number (and percentage) of PCV-3 qPCR positive pools (quantifiable and non-quantifiable) of tissues from fetuses of different gestational ages and groups.

Origin	Second Gestational Third		Last Gestational Third		Total of qPCR Positive Pools (%)
	Proportion of qPCR Positive Pools (%)	Number and Mean (Min–Max) PCV-3 Viral Load of Positive and Quantifiable Pools (PCV-3 DNA Copies/mL)	Proportion of qPCR Positive Pools (%)	Number and Mean (Min–Max) PCV-3 Viral Load of Positive and Quantifiable Pools (PCV-3 DNA Copies/mL)	
**NRP**	1/3 (33.3%)	BQL *	68/246 (27.6%)	62 1.49 × 10^8^ (1.16 × 10^3^–3.06 × 10^9^)	69/249 (27.7%)
**S**	4/46 (8.7%)	1 1.07 × 10^6^ (1.07 × 10^6^)	1/5 (20%)	1 9.33 × 10^5^ (9.33 × 10^5^)	5/51 (9.8%)
**Total**	5/49 (10.2%)	1 1.07 × 10^6^ (1.07 × 10^6^)	69/251 (27.5%)	63 1.46 × 10^8^ (1.16 × 10^3^–3.06 × 10^9^)	74/300 (24.6%)

NRP: no reproductive problems; S: slaughterhouse. * BQL: below quantification limit.

**Table 2:** Number (and percentage) of PCV-3 qPCR positive tissue samples (quantifiable and non-quantifiable) from fetuses of the last third of gestation.

Tissue	Last Gestational Third	
	Proportion of qPCR Positive Samples (%)	Number and Mean (Min–Max) PCV-3 Viral Load of Positive and Quantifiable Samples (PCV-3 DNA Copies/mL)
**Brain**	49/64 (76.5%)	48; 1.68 × 10^8^ (2.42 × 10^3^–3.06 × 10^9^)
**Heart**	7/9 (77.7%)	6; 5.41 × 10^7^ (3.36 × 10^4^–1.54 × 10^8^)
**Lung**	51/59 (86.4%)	51; 5.82 × 10^8^ (1.16 × 10^3^–7.61 × 10^9^)
**Kidney**	2/4 (50%)	1; 2.40 × 10^6^
**Spleen**	7/8 (100%)	7; 7.31 × 10^6^ (2.48 × 10^3^–4.91 × 10^7^)

## Data Availability

Data of this study is available upon request to the authors.
